# Botulinum toxin A decreases neural activity in pain-related brain regions in individuals with chronic ocular pain and photophobia

**DOI:** 10.3389/fnins.2023.1202341

**Published:** 2023-06-19

**Authors:** Nicholas Reyes, Jaxon J. Huang, Anjalee Choudhury, Nicholas Pondelis, Elyana V. Locatelli, Elizabeth R. Felix, Pradip M. Pattany, Anat Galor, Eric A. Moulton

**Affiliations:** ^1^Surgical Services, Miami Veterans Administration Medical Center, Miami, FL, United States; ^2^Bascom Palmer Eye Institute, University of Miami, Miami, FL, United States; ^3^Brain and Eye Pain Imaging Lab, Pain and Affective Neuroscience Center, Department of Anesthesia, Critical Care and Pain Medicine, Boston Children’s Hospital and Harvard Medical School, Boston, MA, United States; ^4^Research Service, Miami Veterans Administration Medical Center, Miami, FL, United States; ^5^Physical Medicine and Rehabilitation, University of Miami, Miami, FL, United States; ^6^Department of Radiology, University of Miami, Miami, FL, United States; ^7^Department of Ophthalmology, Boston Children’s Hospital and Harvard Medical School, Boston, MA, United States

**Keywords:** ocular pain, fMRI, pain processing, botulinum toxin A (BoNT-A), photophobia

## Abstract

**Introduction:**

To examine the effect of botulinum toxin A (BoNT-A) on neural mechanisms underlying pain and photophobia using functional magnetic resonance imaging (fMRI) in individuals with chronic ocular pain.

**Methods:**

Twelve subjects with chronic ocular pain and light sensitivity were recruited from the Miami Veterans Affairs eye clinic. Inclusion criteria were: (1) chronic ocular pain; (2) presence of ocular pain over 1 week recall; and (3) presence of photophobia. All individuals underwent an ocular surface examination to capture tear parameters before and 4–6 weeks after BoNT-A injections. Using an event-related fMRI design, subjects were presented with light stimuli during two fMRI scans, once before and 4–6 weeks after BoNT-A injection. Light evoked unpleasantness ratings were reported by subjects after each scan. Whole brain blood oxygen level dependent (BOLD) responses to light stimuli were analyzed.

**Results:**

At baseline, all subjects reported unpleasantness with light stimulation (average: 70.8 ± 32.0). Four to six weeks after BoNT-A injection, unpleasantness scores decreased (48.1 ± 33.6), but the change was not significant. On an individual level, 50% of subjects had decreased unpleasantness ratings in response to light stimulation compared to baseline (“responders,” *n* = 6), while 50% had equivalent (*n* = 3) or increased (*n* = 3) unpleasantness (“non-responders”). At baseline, several differences were noted between responders and non-responders; responders had higher baseline unpleasantness ratings to light, higher symptoms of depression, and more frequent use of antidepressants and anxiolytics, compared to non-responders. Group analysis at baseline displayed light-evoked BOLD responses in bilateral primary somatosensory (S1), bilateral secondary somatosensory (S2), bilateral anterior insula, paracingulate gyrus, midcingulate cortex (MCC), bilateral frontal pole, bilateral cerebellar hemispheric lobule VI, vermis, bilateral cerebellar crus I and II, and visual cortices. BoNT-A injections significantly decreased light evoked BOLD responses in bilateral S1, S2 cortices, cerebellar hemispheric lobule VI, cerebellar crus I, and left cerebellar crus II. BoNT-A responders displayed activation of the spinal trigeminal nucleus at baseline where non-responders did not.

**Discussion:**

BoNT-A injections modulate light-evoked activation of pain-related brain systems and photophobia symptoms in some individuals with chronic ocular pain. These effects are associated with decreased activation in areas responsible for processing the sensory-discriminative, affective, dimensions, and motor responses to pain.

## 1. Introduction

Ocular pain is a frequently encountered symptom in the eye care clinic. It is often included under the umbrella term dry eye (DE), a term which comprises a number of symptoms and signs. Some individuals describe their pain with qualifiers like “dryness,” “foreign body sensation,” and “scratchiness” and in many, objective abnormalities, such as poor tear production, tear film instability, and/or epithelial disruption, are noted on exam. Other individuals characterize their ocular symptoms as “burning,” and report increased pain with exposure to wind and light. These specific complaints are often associated with symptom severity that is out of proportion to objective metrics of tear dysfunction (Moshirfar et al., 2022). In fact, the symptom profile reported by the latter group has similarities to chronic neuropathic pain disorders, and thus, neuropathic mechanisms are thought to underlie ocular pain in these patients (Kalangara et al., 2016, 2017; Galor et al., 2018). Given the negative impact that ocular pain symptoms have on physical and mental functioning (Pouyeh et al., 2012) and the fact that pain persists in many individuals despite therapies aimed at improving ocular surface health (Ames and Galor, 2015; Galor et al., 2016; Lollett and Galor, 2018), new treatments are needed that effectively target the neuropathic mechanisms underlying these symptoms (Lee et al., 2022).

The variability of symptom presentation and of ocular surface exam findings suggest that chronic ocular pain can be driven by both nociceptive and neuropathic mechanisms. Nociceptive sources of pain, including inflammation and epithelial disruption, can be detected with point of care tests or slit lamp examination and treated with topical therapies geared toward improving ocular surface conditions (Ames and Galor, 2015; Galor et al., 2016; Lollett and Galor, 2018). Neuropathic pain can arise from abnormalities anywhere along the trigeminal pain pathway, including within corneal nerves, the trigeminal nuclei, thalamus, and/or higher cortical structures (Rosenthal and Borsook, 2012; Choudhury et al., 2023). Tools that have been used to examine nerve structure and function as they relate to ocular pain include *in-vivo* confocal microscopy (IVCM) and Belmonte and Cochet-Bonnet esthesiometers, respectively (Aykut et al., 2018; Ross et al., 2020; Alabi and Simpson, 2022). With respect to the central nervous system, functional magnetic resonance imaging (fMRI) has been used to examine neural mechanisms that underlie ocular pain, along with other chronic pain conditions (Huang et al., 2011; Cucchiara et al., 2015; Schulte et al., 2018; Xu et al., 2020; Kim et al., 2021; Choudhury et al., 2023). While many advanced techniques now exist, currently, clinical evaluation remains the primary method for detecting a neuropathic component of ocular pain; clinical diagnosis is based on a composite of findings including: (1) pain out of proportion to clinical findings (Ong et al., 2018); (2) ocular pain characterized as “burning” (Kalangara et al., 2017); (3) evoked pain to wind and/or light (Kalangara et al., 2017); (4) abnormal corneal sensitivity and/or anatomy (Galor et al., 2021); (5) persistent symptoms after application of a topical anesthetic (Crane et al., 2017); (6) periocular cutaneous allodynia; and/or (7) the presence of co-morbid pain conditions, such as migraine and fibromyalgia (Galor et al., 2016; Vehof et al., 2016; Farhangi et al., 2020).

In particular, photophobia, or evoked pain to light, is a key predictor of changes in central nociceptive processing in individuals with ocular pain (Rodriguez et al., 2022). This use of photophobia as a diagnostic is further supported by evidence of light-induced activation of the trigemino-cortical pathway in individuals with photophobia and chronic ocular pain (Choudhury et al., 2023). While central nociceptive changes exist within subsets of patients with chronic ocular pain, few have published on its treatment. Data suggest that systemic strategies used in the treatment of neuropathic pain conditions outside the eye can be successfully applied to the treatment of ocular pain (Dieckmann et al., 2017; Jacobs, 2017; Small et al., 2020; Venkateswaran et al., 2020; Mehra et al., 2021; Patel et al., 2021).

There is growing interest in examining botulinum toxin A (i.e., BoNT-A) treatment for neuropathic pain conditions (Egeo et al., 2020), particularly as a therapy for neuropathic ocular pain based on its use in migraine (Diel et al., 2020; Baksh et al., 2021). In an open label study of 76 individuals with chronic migraine, BoNT-A improved symptoms of photophobia measured by Visual Light Sensitivity Questionnaire-8 (VLSQ-8) scores pre-injection vs. post-injection (29.8 vs. 27.7; *p* = 0.002) (Diel et al., 2019). BoNT-A has also been studied outside the purview of migraine: in a case series of 4 individuals with neuropathic ocular pain, BoNT-A improved both pain and light sensitivity in all 4 patients measured by pre-injection versus post-injection VLSQ-8 (mean difference = −10.5) and Dry Eye Questionnaire-5 (DEQ-5) (mean difference = −8) scores. These results highlight the need for more studies that examine precision-based therapies targeting putative neuropathic mechanisms in individuals with chronic ocular pain. To bridge this knowledge gap, we used functional neuroimaging to investigate neural mechanisms underlying chronic ocular pain before and after BoNT-A injection.

## 2. Materials and methods

### 2.1. Standard protocol approvals, registrations, and patient consents

The study was approved by the Miami Veterans Affairs (VA) and the University of Miami Institution Review Boards (IRB approval #3011.08 and 20,190,340, respectively). The study was conducted in accordance with the principles of the Declaration of Helsinki and complied with the requirements of the United States Health Insurance Portability and Accountability Act. Written informed consent was obtained from all participants prior to any study activities.

### 2.2. Study population

Subjects were recruited from the Miami VA eye clinic if they presented with chronic (≥3 months) ocular pain (average ocular pain over 1 week recall ≥1, scale 0–10) and light sensitivity (Neuropathic Pain Symptom Inventory modified for the Eye, question #9 ≥ 1) (Schiffman et al., 2000; Farhangi et al., 2019). Ocular pain symptoms were characterized using several descriptors, such as “burning,” “aching,” “splitting,” and “piercing.” Baseline data from six patients in the current cohort were also used in a previous analysis investigating photophobia in individuals with chronic ocular pain using fMRI (Choudhury et al., 2023). Exclusion criteria included ocular diseases that could confound photophobia symptoms such as glaucoma, use of glaucoma medications, uveitis, iris transillumination defects, retinal degeneration, and anatomic abnormalities of the cornea, conjunctiva, or eyelids. Individuals were also excluded if they did not report symptoms (e.g., “burning”) or display clinical exam findings (e.g., abnormal corneal sensitivity and/or anatomy, persistent symptoms after application of topical anesthetic, periocular cutaneous allodynia, co-morbid pain conditions) supportive of neuropathic ocular pain. Additionally, we excluded individuals with contraindications to fMRI scanning (e.g., pregnancy, pacemaker, implanted metal device). No subjects were excluded based on medication use or the presence of a systemic condition.

### 2.3. Study design

A pre-post treatment study design was used. A baseline clinical exam visit collected tear parameters and questionnaire data. fMRI scans were performed between 1 week before or up to 4 weeks after the initial clinical exam. BoNT-A injections were then administered between 1 and 13 weeks after completing all baseline assessments. Post-treatment assessments were performed 4–6 weeks after BoNT-A injection in the same manner as at baseline/pre-treatment.

### 2.4. Questionnaires

During the baseline clinical visit, subjects were administered questionnaires to collect demographic and supporting health information. All individuals also filled out standardized questionnaires regarding ocular symptoms pre-BoNT-A, including the 5 item Dry Eye Questionnaire 5 (DEQ-5, range 0–22) (Chalmers et al., 2010), the Ocular Surface Disease Index (OSDI, range 0–100), a Numerical Rating Scale (NRS) for average ocular pain intensity during the past week (range 0–10) (Schiffman et al., 2000), Patient Health Questionnaire-9 (PHQ-9, range 0–27) (Kroenke et al., 2001), and the Neuropathic Pain Symptom Inventory modified for the Eye (NPSI-Eye, range 0–100) (Farhangi et al., 2019). Ten individuals had ocular symptoms data from the clinical visit post-BoNT-A (one individual had a post BoNT-A fMRI but no follow-up clinic visit, while another did not fill out questionnaires during their second clinic visit).

### 2.5. Ocular surface evaluation

At baseline and 4–6 weeks post BoNT-A, subjects underwent a clinical ocular surface evaluation, including tear breakup time (TBUT) (measured in seconds, with lower values indicating less tear stability), fluorescein corneal staining (graded to the National Eye Institute (NEI) scale (Wolffsohn et al., 2017) with higher values indicating a more irregular epithelium), and tear production using anesthetized Schirmer strips test (measured in millimeters of wetting in 5 min, with lower values indicating less tear production). This was completed for all individuals pre-BoNT-A and 11 individuals post-injection.

### 2.6. Botulinum toxin protocol

A modified migraine protocol (Venkateswaran et al., 2020) using botulinum toxin A (Botox, Allergan) was administered to all subjects, irrespective of concomitant ocular surface treatments, at 7 sites on the forehead, for a total of 35 units: 5 units in the procerus, 10 units in the corrugators, and 20 units in the frontalis.

### 2.7. fMRI protocol

The fMRI protocol was adopted and modified from a prior study on photophobia using visual stimuli to evoke pain and identify trigeminal nociceptive and other pain-related pathways (Moulton et al., 2009). fMRI acquisition and pre-processing steps for whole brain analysis are included in Supplementary Text 1. All subjects underwent two fMRI sessions, first before BoNT-A injection and again 4–6 weeks after injection. During each session, individuals were presented with intermittent bright light in a darkened environment. The presentation consisted of two visual conditions: a black screen rest condition, which featured a white fixation cross on a black background (~0.5 lux); and a white screen stimulus condition, which featured a black fixation cross on a white background (~65 lux). Subjects were presented with 16 episodes of sustained bright light (white screen), each lasting 6 s. To avoid anticipatory processes, the inter-stimulus interval (ISI) varied between 26 and 34 s in 2-s increments. The scanner environment was kept dark during the entire experiment, with only a projector providing intermittent brief illumination. Each fMRI scan was performed immediately following placement of a single eye drop of artificial tears (Refresh Plus Lubricant Eye Drops, Allergan) in each eye. Subjects were instructed to keep eyes open and blink normally throughout the duration of each scan.

### 2.8. fMRI screen condition ratings

At the end of the 16 stimulus presentations, subjects rated their sensation of unpleasantness experienced when viewing the light stimulus condition (white screen) and the rest condition (black screen). Unpleasantness ratings were reported verbally via the MRI scanner microphone, while the participant was still inside the bore, using a numerical rating scale of 0 (“not unpleasant at all”) to 100 (“most unpleasant sensation imaginable”). Subjects were read instructions regarding use of the rating scale prior to entering the scanner, and any questions or were addressed at that time.

### 2.9. Statistical analysis

Statistical analyses were performed using SPSS V.28.0 (SPSS, Chicago, IL, USA) statistical package. In pre- and post-injection conditions, significant differences in unpleasantness ratings to black and white screen stimuli were analyzed using paired t-tests. In pre- and post-injection conditions comparing those who responded favorably to BoNT-A (responders) and those who did not (non-responders), significant differences were analyzed using independent *t*-tests or Chi-squared tests, as appropriate.

The statistical significance for whole brain group-level contrast analyses was set at a cluster-level threshold of *p* < 0.05. Significant clusters were identified by region, and parameter estimate (PE) values of activation magnitude from each subject were extracted from significant voxels within each region. The PEs across all significant cluster-based voxels of a given region were averaged for each subject. Using Pearson correlation coefficients, averaged PE values were compared against pre-injection PE scores.

Except where otherwise indicated, means are reported with standard deviation (M ± SD).

## 3. Results

### 3.1. Subjects

Twelve individuals with chronic ocular pain and light sensitivity were enrolled into the study. The demographics, comorbidities, and medication use of all subjects are summarized in Table 1. Questionnaire data assessing dry eye, light sensitivity, and ocular pain symptoms and tear metrics are shown in Table 2.

**Table 1:** Demographics, comorbidities, and medication use of subjects.

Demographics	
Age (mean ± SD; years)	53.5 ± 9.6
Sex, male % (subject #)	33.3% (4,5,8,11)
Race, white % (subject #)	83.3% (2–10, 12)
Ethnicity, Hispanic % (subject #)	66.7% (2–5, 7–10)
Self-reported comorbidities	
Diabetes mellitus % (subject #)	0% (0)
PTSD % (subject #)	16.7% (2, 11)
Depression % (subject #)	58.3% (1–5, 7, 11)
Arthritis % (subject #)	25% (2, 6, 8)
Sleep apnea % (subject #)	41.7% (4, 5, 8, 9, 11)
Migraine % (subject #)	25% (1, 2, 6*)
Traumatic brain injury % (subject #)	16.7% (6, 8)
Past or current smoker % (subject #)	33.3% (4, 5, 8, 11)
Self-reported medication use	
Anti-depressant (subject #)	58% (1–6, 11)
Anxiolytic (subject #)	58% (1–6, 11)
Analgesic (subject #)	83% (1, 2, 3–11)
Gabapentin (subject #)	33% (3–6, 8)
Pregabalin (subject #)	8% (2)

**Figure 1:**
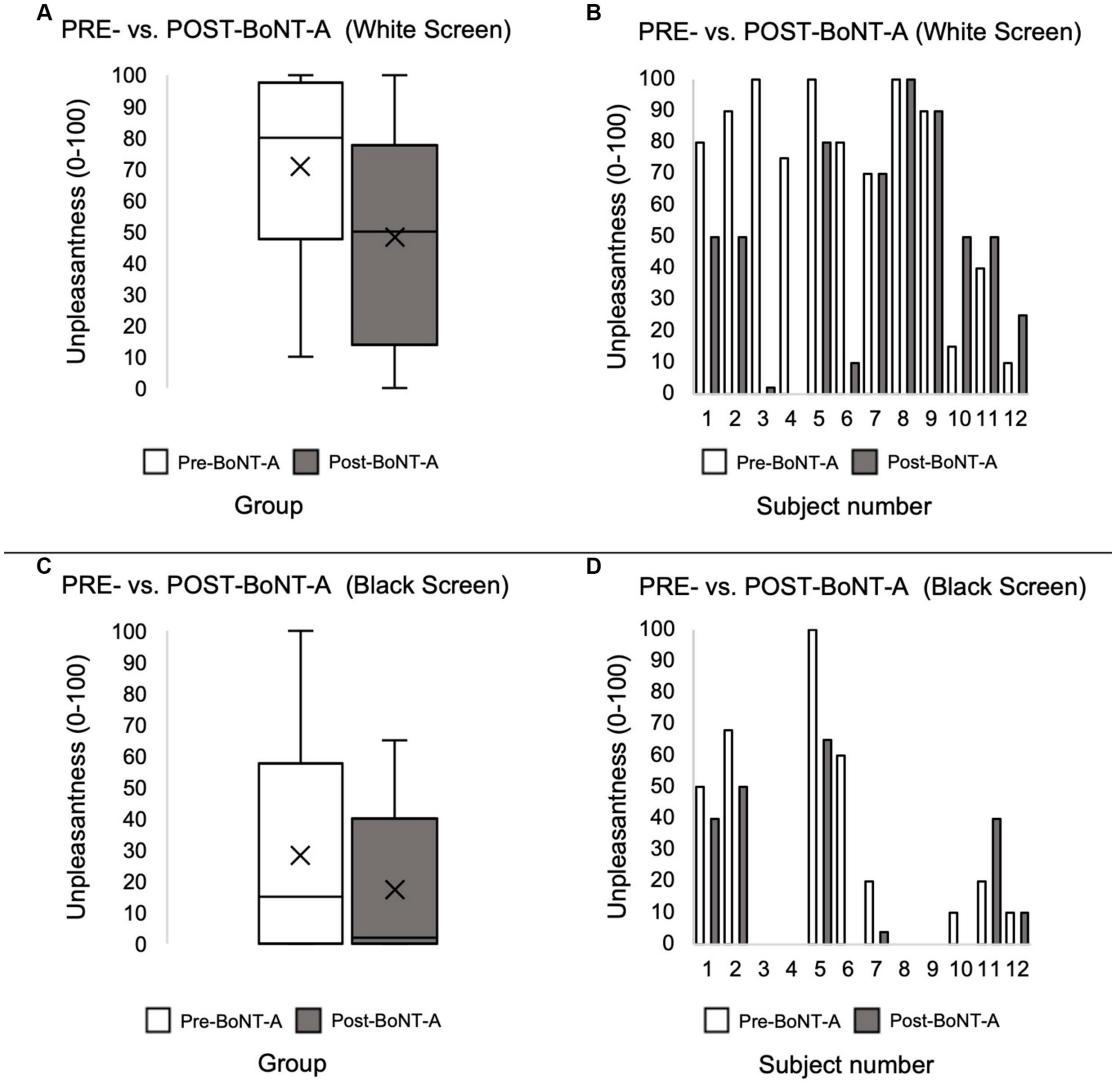
Light stimulus-induced unpleasantness ratings before and after BoNT-A injections at the group level **(A)** and at the individual subject level **(B)**. Rest condition unpleasantness levels before and after BoNT-A injections at the group level **(C)** and at the individual level **(D)**. Unpleasantness ratings were compared at the group level using paired *t*-test.

Subject numbers refer to unique subject identifiers to link patient demographics in the table to unpleasantness reports (Figure 1) and parameters of brain activity (Tables 3, 4). SD, standard deviation; PTSD, post-traumatic stress disorder. *Subject #6 reported active migraine during fMRI scan.

**Table 2:** Ocular symptoms and signs of subjects pre-BoNT-A and post-BoNT-A.

	Pre-BoNT-A	Post-BoNT-A	Value of *p*
DE, light sensitivity, and ocular pain symptoms assessed via questionnaires, mean ± SD (*n*)			
DEQ5 (range 0–22)	15.8 ± 4.3 (12)	14.6 ± 4.1 (11)	0.22
Light sensitivity (OSDI-Q1) (range 0–4)	3.6 ± 0.67 (12)	3.2 ± 1.2 (10)	0.34
OSDI total (range 0–100)	89.6 ± 16.7 (12)	62.54 ± 25.1 (10)	0.53
Light sensitivity (NPSI-Eye-Q9) (range 0–10)	6.3 ± 3.4 (12)	5.9 ± 3.5 (10)	0.89
NPSI-Eye total (range 0–100)	42.5 ± 17.9 (12)	33.5 ± 14.6 (10)	0.04*
Average pain rating 1 week recall (range 0–10)	6 ± 2.5 (12)	4.1 ± 1.7 (10)	0.06
Tear parameters (value taken from the more abnormal eye)			
TBUT (mean ± SD; seconds) (*n*)	6.9 ± 5.0 (12)	6.6 ± 2.4 (11)	0.82
Staining (mean ± SD; range 0–3) (*n*)	1.9 ± 3.9 (12)	2.3 ± 3.4 (11)	0.75
Schirmer’s (mean ± SD; mm) (*n*)	11.3 ± 5.9 (12)	13.8 ± 10.1 (10)	0.31

BoNT-A, botulinum toxin – A; DE, dry eye; SD, standard deviation; n, number of subjects; DEQ5, 5 Item Dry Eye Questionnaire; OSDI, Ocular Surface Disease Index; NPSI-Eye, Neuropathic Pain Symptom Inventory modified for the Eye; TBUT, tear break-up time. *Paired *t*-test, *p* < 0.05.

### 3.2. Change in subjective response and tear parameters after BoNT-A injection

Ocular symptoms were collected on questionnaires before and 4–6 weeks after BoNT-A injections (Table 2). All dry eye, light sensitivity, and ocular pain questionnaire scores improved on a group level 4–6 weeks after BoNT-A injection, with significant improvement on NPSI-Eye total [42.5 ± 17.9 vs. 33.9 ± 14.6, paired *t*-test *t*(9) = −2.46, *p* = 0.04]. On clinical exam, no significant changes were noted with respect to tear film parameters pre vs. post-BoNT-A.

In the pre-BoNT-A condition, unpleasantness ratings for the light stimulus (white screen) were significantly greater than for the rest condition (black screen) [70.8 ± 32.0 vs. 28.2 ± 33.3, paired *t*-test *t*(11) = −3.80, *p* = 0.003], supporting the presence of light sensitivity in our population. Four to six weeks after BoNT-A injections, unpleasantness ratings to the light stimulus decreased compared to before BoNT-A injections, but the difference was not statistically significant [70.8 ± 32.0 vs. 48.1 ± 33.6, paired *t*-test *t*(11) = −1.92, *p* = 0.08] (Figures 1A,B). Half of the participants (*n* = 6) reported decreases, three reported increases, and three reported no change (Figure 1B). No statistical difference was detected in unpleasantness ratings to the rest condition (black screen) before vs. after BoNT-A injections [28.2 ± 33.3 vs. 17.4 ± 24.1, paired *t*-test *t*(11) = −1.82, *p* = 0.10] (Figures 1C,D).

### 3.3. Light-induced fMRI activity in subjects with chronic ocular pain and light sensitivity pre- and post-BoNT-A injections

**Figure 2:**
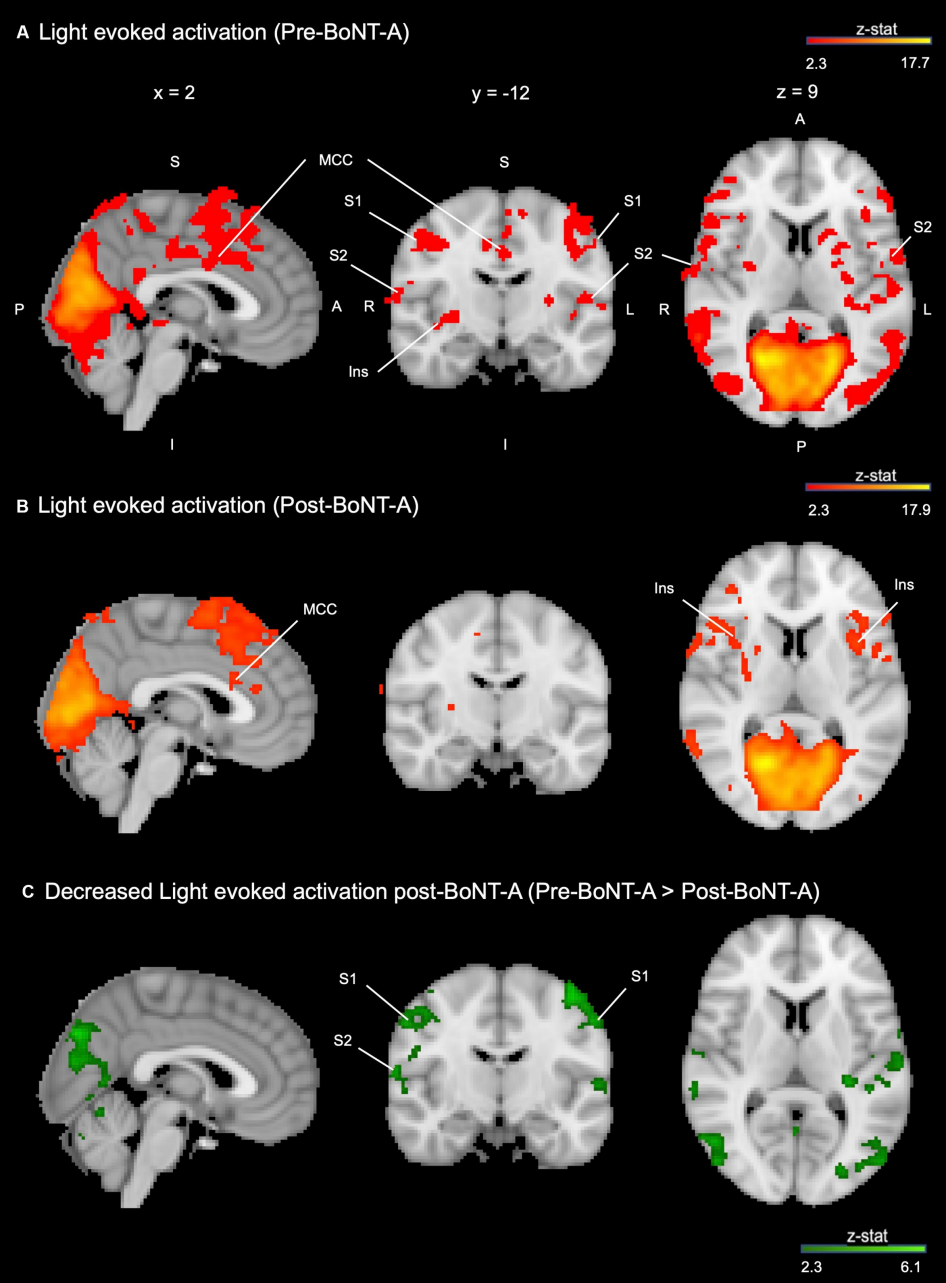
Light-induced activation in the whole brain is decreased 4–6 weeks after BoNT-A injections. **(A)** Group average activation when observing a light stimulus vs. rest condition before BoNT-A injections (red-orange). **(B)** Group average activation when observing a light stimulus vs. rest condition 4–6 weeks after BoNT-A injections (red-orange). **(C)** Group contrast (pre-BoNT-A > post-BoNT-A) displayed with MNI atlas underlay (dark green-light green). Both activation and contrast maps had an individual voxel threshold of *z* > 2.3, and cluster-threshold of *p* < 0.05. S, superior; I, inferior; A, anterior; P, posterior; R, right; L, left; MCC, mid-cingulate cortex; Ins, insula; S1, primary somatosensory cortex; S2, secondary somatosensory cortex.

**Figure 3:**
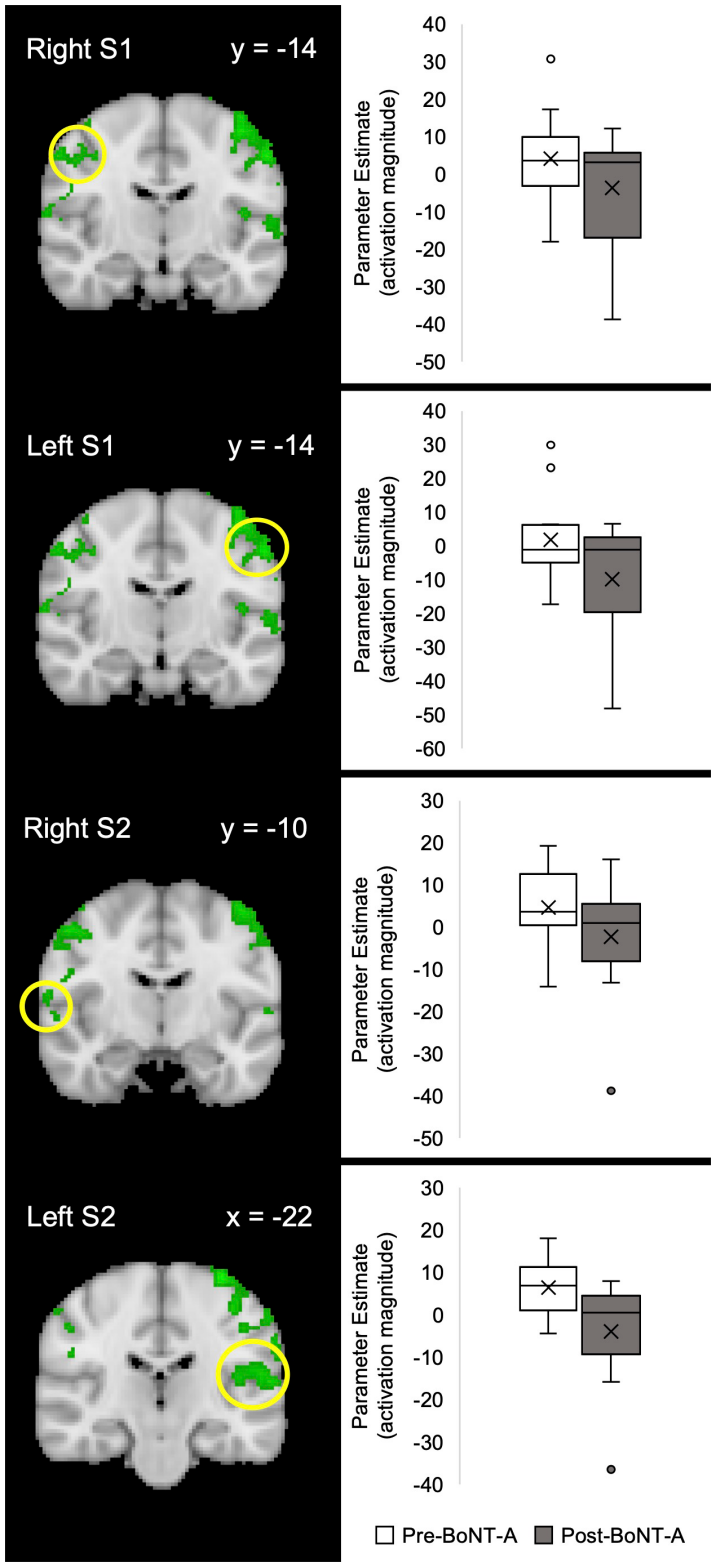
Parameter estimates of fMRI activation pre-BoNT-A vs. post-BoNT-A injections. The Harvard-Oxford Subcortical and Cortical atlases were used to create anatomical masks of each region. BOLD signal activity in response to light for each region of interest is indicated by a yellow circle. Functional masks were created from group-level contrast maps to pull parameter estimates of BOLD signal responses. Contrast maps had an individual voxel threshold of *z* > 2.3, and cluster-threshold of *p* < 0.05. S1, primary somatosensory cortex; S2, secondary somatosensory cortex.

On group analysis, light-induced BOLD responses differed in subjects before and 4–6 weeks after BoNT-A injections. Before BoNT-A injections, subjects had significant BOLD responses to the light stimulus in bilateral primary somatosensory (S1) and secondary somatosensory (S2) cortices, bilateral anterior insula, paracingulate gyrus, and midcingulate cortex (MCC), bilateral frontal pole, bilateral cerebellar hemispheric lobule VI, vermis, bilateral cerebellar crus I and II, and visual cortices (Figure 2A). Four to six weeks after BoNT-A injections, significant BOLD responses to light were detected in the right S1, bilateral anterior insula, MCC, paracingulate, and frontal pole as well as bilateral cerebellar lobule VI, bilateral crus I, left crus II, and bilateral visual cortices (Figure 2B). When comparing pre- vs. post-BoNT-A injections (Figure 2C, Pre-BoNT-A > Post-BoNT-A, and Figure 3), significant decreases in BOLD responses to light stimuli 4–6 weeks after BoNT-A injections were seen in bilateral S1 and bilateral S2 cortices (Supplementary Table S1). Similarly, significantly decreased parameter estimates were found in cerebellar regions in post-BoNT-A compared to pre-BoNT-A conditions, specifically in bilateral crus I, bilateral crus II, bilateral lobule VI, and vermis.

Although decreased cortical activity was observed at the group level, changes in BOLD response to light stimuli were not uniform on an individual subject level. Table 3 describes whether significant BOLD activity in brain regions related to pain processing were present prior to BoNT-A injections (indicated as Yes or No). Table 4 displays changes in BOLD signal response (PEs) in subjects pre-BoNT-A and 4–6 weeks post-BoNT-A injections, as well as changes in evoked unpleasantness scores. In all cortical and cerebellar regions specified, correlations between parameter estimates and unpleasantness ratings were not significant.

**Table 3:** Significant pre-BoNT-A BOLD activity per brain region of interest on the subject level.

Subject (sub-group)	S1 Rt	S1 Lt	S2 Rt	S2 Lt	FP Rt	FP Lt	TP Rt	TP Lt	Ins Rt	Ins Lt	PCG	M C C	P C N	C Crus 1 Rt	C Crus 1 Lt	C Crus 2 Rt	C Crus 2 Lt	C 6 Rt	C 6 Lt	VMS	SpV
1 (R)	N	N	N	N	Y	N	Y	N	Y	N	Y	Y	Y	N	N	N	N	N	N	N	N
2 (R)	N	N	N	N	N	N	N	Y	N	N	N	N	N	Y	N	N	N	Y	N	N	N
3 (R)	Y	Y	Y	Y	Y	Y	Y	Y	Y	Y	Y	Y	Y	Y	Y	Y	Y	Y	Y	Y	Y
4 (R)	N	N	N	N	Y	N	N	N	N	N	Y	Y	Y	Y	Y	N	Y	Y	Y	N	N
5 (R)	Y	Y	Y	Y	Y	Y	Y	Y	Y	Y	Y	Y	N	Y	N	N	N	Y	N	Y	Y
6 (R)	N	N	N	Y	Y	Y	N	Y	N	Y	N	N	Y	N	Y	N	Y	N	N	N	N
7 (NR)	N	Y	N	Y	N	N	N	Y	N	Y	N	Y	Y	Y	Y	N	Y	Y	Y	Y	N
8 (NR)	N	N	N	N	N	N	N	Y	N	N	N	N	Y	N	N	N	N	N	N	N	N
9 (NR)	Y	Y	N	N	Y	Y	Y	Y	Y	Y	Y	Y	Y	Y	Y	Y	Y	Y	Y	Y	N
10 (NR)	Y	N	Y	N	N	Y	N	N	N	Y	Y	N	Y	Y	Y	N	N	Y	Y	N	N
11 (NR)	Y	Y	Y	Y	Y	Y	Y	Y	Y	Y	Y	Y	Y	Y	Y	Y	Y	Y	Y	Y	Y
12 (NR)	N	N	N	N	Y	N	N	N	N	N	Y	Y	Y	N	N	N	N	Y	Y	N	N

Rt, right; Lt, left; S1, primary somatosensory cortex; S2, secondary somatosensory cortex; FP, frontal pole; TP, temporal pole; Ins, insula; PCG, paracingulate; MCC, mid-cingulate cortex; PCN, precuneous; C Crus 1, cerebellar crus 1; C Crus 2, cerebellar crus 2; C 6, cerebellar hemispheric lobule VI; VMS, vermis; SpV, spinal trigeminal nucleus; R, responder; NR, non-responder; Y, significant BOLD activity in brain region of interest; N, no significant BOLD activity in brain region of interest.

**Table 4:** Subject-level change in BOLD activity and unpleasantness scores in response to light stimulus pre-BoNT-A vs. post-BoNT-A.

Subject (subgroup)	∆ S1 Rt	∆ S1 Lt	∆ S2 Rt	∆ S2 Lt	∆ C Crus 1 Rt	∆ C Crus 1 Lt	∆ C Crus 2 Lt	∆ C 6 Rt	∆ C 6 Lt	∆ vermis	∆ SpV	∆ Unpls
1 (R)	−35.2	−47.7	−45.6	−42.9	0.2	2.9	−3.6	−15.5	−8.9	−11.8	−3.6	**↓**
2 (R)	30.1	23.8	8.1	5.3	−6.8	−17.5	−17.9	−1.7	2.6	0.1	−15.6	**↓**
3 (R)	−27.8	−28.2	−23.2	−14.2	−3.2	−18.8	−17.8	−26.0	−32.6	−22.2	−11.4	**↓**
4 (R)	5.6	9.7	−0.3	−3.8	7.1	−5.1	0.3	9.2	9.4	7.7	−5.0	**↓**
5 (R)	−0.4	−22.5	−15.5	−14.5	9.4	11.9	10.3	4.5	13.2	10.2	−19.9	**↓**
6 (R)	8.3	−22.6	−8.7	−22.7	0.7	−23.3	−11.8	0.1	−1.9	−2.0	1.9	**↓**
7 (NR)	7.9	−4.6	12.1	−11.4	0.1	8.6	11.3	−0.7	9.0	1.8	6.6	No change
8 (NR)	−24.2	−14.9	−21.6	−14.5	−9.5	−14.5	0.1	−6.1	−7.2	2.3	8.8	No change
9 (NR)	−15.9	−1.0	16.2	12.2	1.3	−19.0	−24.6	−9.3	−15.4	−2.9	25.2	No change
10 (NR)	−25.9	−20.5	−10.2	−10.9	−2.9	−2.0	−6.2	−19.6	−5.2	−6.0	26.0	**↑**
11 (NR)	−17.3	−7.7	−7.4	−8.3	−12.7	−15.1	−11.7	−24.8	−23.4	−22.5	−0.4	**↑**
12 (NR)	0.5	−3.6	14.0	1.5	3.5	1.0	−7.9	9.6	6.7	2.2	9.0	**↑**

R, responder; NR, non-responder; S1, primary somatosensory cortex; S2, secondary somatosensory cortex; C, cerebellar; C6, cerebellar hemispheric lobule VI; SpV, spinal trigeminal nucleus; Unp, unpleasantness.

### 3.4. Clinical and fMRI findings in BoNT-A responders and non-responders

**Figure 4:**
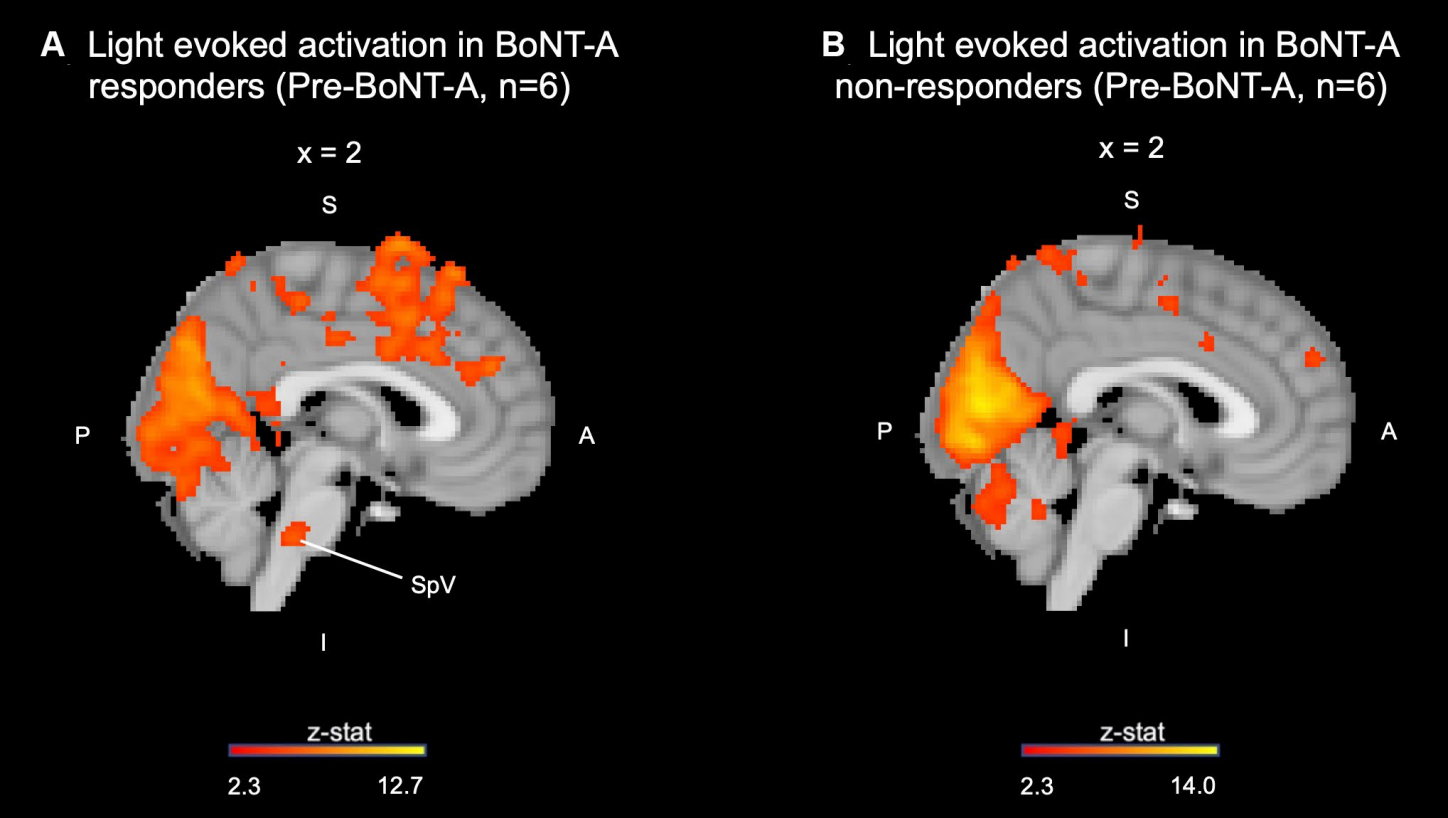
Pre-BoNT-A light induced activation in the spinal trigeminal nucleus present in **(A)** BoNT-A responders and not in **(B)** non-responders. SpV, spinal trigeminal nucleus; S, superior; I, inferior; A, anterior; P, posterior.

Subjects were divided into two groups based on clinical response to BoNT-A. Group 1 was comprised of individuals who reported decreased unpleasantness scores to the light stimuli 4–6 weeks post-BoNT-A injections [responders, *n* = 6, 87.5 ± 10.8 vs. 32 ± 32.7, paired *t*-test *t*(5) = −4.51, *p* = 0.006]. Group 2 reported no change (*n* = 3) or worsening (*n* = 3) in unpleasantness scores after light stimulus post-BoNT-A [non-responders, *n* = 6, 54.2 ± 38.3 vs. 64.2 ± 28.0, paired *t*-test *t*(5) = 1.78, *p* = 0.136]. Clinical factors that differed between groups included the presence of at least mild depression [PHQ-9 ≥ 5 vs. <5, χ^2^ (1, *n* = 12) =8.57, *p* = 0.003], use of anti-depressants [χ^2^ (1, *n* = 12) =8.57, *p* = 0.003], and use of anxiolytics [χ^2^ (1, *n* = 12) = 8.57, *p* = 0.003] in responders. No significant differences in tear film parameters were found between the two groups. Imaging revealed that individuals who responded to BoNT-A had spinal trigeminal nucleus (SpV) activation to light stimulus prior to BoNT-A injections, while this finding was absent in non-responders (Figure 4). This observation highlights a potentially key objective difference in pain processing pathways between BoNT-A responders and non-responders; the activity of SpV during photophobia may predict the success of BoNT-A injections as a therapy for individuals suffering from chronic ocular pain.

## 4. Discussion

In this study, we found that BoNT-A modulated responses to light in some individuals with chronic ocular pain and photophobia. As a group, questionnaires reflected improved ocular pain and photophobia symptoms after BoNT-A injections. However, variability was noted on an individual level. Group level fMRI analysis showed that BoNT-A injections significantly decreased light-induced brain activity in regions related to pain processing, most notably in bilateral S1, S2, hemispheric lobule VI, and crus I as well as left crus II, and cerebellar vermis. This indicates that BoNT-A injections may modulate subjective symptom improvement as these changes were concurrently detected in brain regions responsible for affective, sensory-discriminatory, and motor responses in pain processing. Some clinical and fMRI findings differed in individuals who reported improved light tolerance after BoNT-A compared to those who did not have a favorable response. These included the presence of depression symptoms, use of antidepressants and anxiolytics, and spinal trigeminal nucleus (SpV) activation to light stimuli prior to BoNT-A injections. The latter finding suggests that BoNT-A may have enhanced efficacy in individuals with higher light-evoked activity in trigeminal nerve pathways prior to treatment.

### 4.1. Photophobia mechanisms

In patients with chronic ocular pain, photophobia has been found to be a marker for central neuroplasticity (Choudhury et al., 2023). We have previously shown that individuals with ocular pain and neuropathic features, such as photophobia, had a higher frequency of centralized pain conditions (e.g., trigeminal neuralgia, back pain) (Crane et al., 2017), persistent pain after placement of a topical anesthetic (Crane et al., 2017), concomitant depression and anxiety, and decreased quality of life metrics compared to those without neuropathic features (Crane et al., 2017). In support of this, our previous study showed significant light-evoked activation in the SpV, S1 cortex, and other brain regions related to pain processing in individuals with chronic ocular pain and photophobia compared to controls without pain (Choudhury et al., 2023). In addition, we previously demonstrated that self-reported photophobia had a reasonable sensitivity (85%) for the presence of aftersensations, a marker of central sensitization (Rodriguez et al., 2022). Together, these studies suggest that central abnormalities underlie photophobia in individuals with chronic ocular pain.

Multiple photophobia neural circuits have been proposed that integrate light perception with nociceptive and autonomic responses in both animal and human models (Moulton et al., 2009; Okamoto et al., 2009; Noseda et al., 2010; Katagiri et al., 2013; Rahman et al., 2014; Marek et al., 2019). In the trigeminal pain pathway, V1 branches found within the eye and surrounding regions, including orbital vessels, are responsible for transmitting afferent nociceptive signals and autonomic activity. When stimulated by a noxious stimulus, afferent nerve fibers within V1 release mediators such as calcitonin gene related peptide and nitric oxide which leads to a signaling cascade to the trigeminal nucleus caudalis (TNC), posterior thalamus, and higher order cortices to produce pain sensations (Digre and Brennan, 2012; Diel et al., 2020). At the group level, we observed activation of a cortical center involved in this pathway (right S1) as well as proximal regions in the brainstem (SpV) within the BoNT-A responders subgroup. Alternatively, BoNT-A non-responders may involve other photophobia neural networks to a greater extent, which may explain why this subgroup did not display significant activation in the trigeminal pathway.

One alternative photophobia pathway involves melanopsin, a photoactive pigment maximally activated at 480 nm wavelengths of light. Populations of these cells are found in intrinsic photosensitive retinal ganglion cells (ipRGCs), named for their unique capability to detect light independently from traditional rod and cone photoreceptors (Berson et al., 2002). Once activated, ipRGCs signal directly to posterior thalamic nuclei, which then project onto cortical regions responsible for pain processing (Noseda et al., 2010). Interestingly, melanopsin-containing cells also have been found in the cornea and iris in animal models (Delwig et al., 2018) and trigeminal ganglion neurons in humans (Matynia et al., 2016), though their functional significance is controversial (Delwig et al., 2018). Though we did not observe activity in the posterior thalamus in our cohort, there is potential for these networks to be interconnected, and melanopsin-activated trigeminal afferents could conceivably lead to pain sensations (Marek et al., 2019). Reports of unpleasantness after therapeutic intervention in our cohort may indicate that the melanopsin pathway is not affected by BoNT-A and still contributes to photophobia.

### 4.2. BoNT-A injection used as treatment for chronic pain conditions related to the head and neck

Though BoNT-A as a treatment for ocular surface diseases is relatively novel (Venkateswaran et al., 2020), it has been used for other conditions related to the head and neck, including migraine (Diel et al., 2019; Shaterian et al., 2022), benign essential blepharospasm (BEB) (Jankovic et al., 2011; Mitsikostas et al., 2020), trigeminal neuralgia (TGN) (Borodic and Acquadro, 2002), and cervical dystonia (Charles et al., 2012; Evidente et al., 2013). As seen in one study of 42 subjects with masseter muscle pain related to temporomandibular joint dysfunction and tension-type headache, BoNT-A injections decreased pain episodes in the temporal region bilaterally as well as pain intensity and analgesic drug intake (Pihut et al., 2016). In support of its use for ocular pain, a case series of 4 patients with migraine and photophobia found that BoNT-A injections improved symptoms of both photophobia and ocular pain (Venkateswaran et al., 2020). In our study, 50% of individuals with chronic ocular pain and photophobia reported improved levels of light-evoked unpleasantness 4–6 weeks after receiving BoNT-A injections.

### 4.3. Pathophysiological mechanisms related to BoNT-A effect on our cohort

BoNT-A may improve symptoms of ocular pain and photophobia through multiple potential mechanisms related to trigeminal nerve pathways. Ocular surface disturbances and light both activate the trigeminal pathway by triggering first-order neurons within the ophthalmic division of the trigeminal nerve (V1) (Diel et al., 2019). The first proposed mechanism of BoNT-A acts at this level of the pathway by decreasing facial muscle contraction, subsequently decreasing stimulation and signaling through primary trigeminal afferents (Diel et al., 2019). Supporting this mechanism are previous studies demonstrating mechanical hyperalgesia and central sensitization mediated by the release of calcitonin gene related peptide (CGRP) after continuous muscle fiber activation (Diel et al., 2019; Venkateswaran et al., 2020). CGRP mediates neurogenic inflammation, and it has been shown to trigger photophobia and migraine by sensitization of trigeminal neurons in both animal and human models (Kaiser et al., 2012; Guo et al., 2016). In addition, CGRP potentiates the effect of other nociceptive neuropeptides, such as substance P and glutamate, increasing pain perception (Venkateswaran et al., 2020). Another mechanism by which BoNT-A may act to decrease CGRP release from trigeminal neurons is through the cleavage of synaptosomal-associated protein 25 (SNAP-25) (Diel et al., 2019). SNAP-25 is a vesicle-docking protein involved in the calcium-regulated neuroexocytosis of neurotransmitters related to light aversion as well as mediators of inflammation and pain, such as CGRP (Diel et al., 2019). By cleaving SNAP-25, BoNT-A inhibits synaptic exocytosis, blocking the neural transmission of these mediators and reducing pain and photophobia (Meng et al., 2009). Therefore, BoNT-A’s action at the neuromuscular junction results simultaneously in decreased muscle contraction and the increased cleavage of SNAP-25, which both reduce CGRP release, and together may explain the improved symptoms of pain and photophobia in our BoNT-A responders (Diel et al., 2019; Venkateswaran et al., 2020).

### 4.4. Photophobia processing beyond the trigeminal system

In addition to acting through the trigeminal pathway, BoNT-A was found to decrease the activation of brain regions associated with various dimensions of pain processing in our study. The dimensions of pain include a sensory-discriminative component, an affective-motivational component, and a motor component (Moulton et al., 2010; Talbot et al., 2019). The sensory-discriminative dimension refers to the intensity and quality of pain, as well as the spatial and temporal characteristics (Talbot et al., 2019). The affective-motivational dimension refers to the overall unpleasantness of the pain, and the motivation that drives actions to prevent and avoid this pain (Talbot et al., 2019). Lastly, the motor component describes the motor response to pain, such as withdrawing from a painful stimulus (Moulton et al., 2010). In our study, group analysis of whole brain activation showed a decrease in light-evoked BOLD signal in the bilateral S1, bilateral S2, bilateral cerebellar hemispheric lobule VI, bilateral cerebellar crus I, left cerebellar crus II, and vermis 4–6 weeks post-BoNT-A injection compared to pre-BoNT-A injection. S1 and S2 are associated with the sensory-discriminative component of pain, whereas the cerebellar vermis is thought to be involved in the motor aspect of pain (Lamm et al., 2007; Diano et al., 2016). In addition, S2 and the posterior cerebellar hemispheres (including lobule VI, crus I, and crus II) are associated with the affective-motivational dimension of pain (Lamm et al., 2007; Moulton et al., 2010, 2011). It is also interesting to note that brain regions such as S1 and the cerebellum have been shown to be activated by the anticipation or expectation of pain in the absence of a pain stimulus (Ploghaus et al., 1999; Porro et al., 2002). Therefore, it is possible that the decreased activation observed in these regions could in part be due to a reduced expectation of light-evoked pain post-BoNT-A injection. Taken together, the roles of each brain region in the different dimensions of pain, along with their involvement within various pain processing pathways, may explain the differing correlations between PEs and evoked unpleasantness found in our study.

### 4.5. Photophobia processing and brain circuits related to mood

In our study, BoNT-A responders more frequently reported mild or greater symptoms of depression and use of anti-depressants and anxiolytics compared to BoNT-A non-responders. Interestingly, BoNT-A has been used as treatment for mood disorders such as major depressive disorder (MDD) (Magid et al., 2014; Parsaik et al., 2016; Wollmer et al., 2022). In addition, ocular pain has been associated with the presence of mood disorders, such as MDD (Galor et al., 2012; Zhou et al., 2022). While there are no previous studies investigating the effect of BoNT-A on fMRI measures in individuals with MDD, one hypothesis by which BoNT-A decreases depressive symptoms is by acting on the amygdala. Specifically, in one fMRI study of individuals without MDD, BoNT-A injection of the facial muscles resulted in decreased activation of the left amygdala during imitation of angry facial expressions (Hennenlotter et al., 2009). It is theorized that the denervation of facial muscles results in reduced afferent sensory signals to the trigeminal tract, brainstem, and left amygdala (Magid et al., 2014). Since hyperactivity of the left amygdala has been associated with MDD and anxiety, BoNT-A’s ability to decrease amygdala activation provides a potential mechanism for its efficacy in treating MDD (Magid et al., 2014). Neuroimaging studies have also been conducted in individuals with MDD at baseline. With resting state fMRI, individuals with MDD have been shown to have increased activity in the insula and superior frontal gyrus (SFG) (including the prefrontal cortex, orbitofrontal cortex, and anterior cingulate cortex), and decreased activation in the precuneus, occipital cortex, and posterior lobes of the cerebellum (mainly lobule VI and crus I), compared to controls (Gong et al., 2020; Siegel-Ramsay et al., 2022). There are parallels between these neuroimaging findings in patients with MDD and our patient cohort. Taken together, our results suggest that BoNT-A injections may serve as targeted therapy for individuals who have chronic ocular pain and photophobia, a history of MDD, and are taking anti-depressants and anxiolytics.

Another area that may be impacted by BoNT-A treatment and is involved in MDD is the cerebellum. The cerebellum has been implicated in both emotional (Adamaszek et al., 2017) and pain processing (Moulton et al., 2010; Adamaszek et al., 2017). Specifically, the cerebellum is involved in emotional perception and functions in circuits that are disrupted in mood disorders, such as MDD (Adamaszek et al., 2017). Additionally, the cerebellum receives ascending and descending nociceptive inputs, and regions including the cerebellar vermis, lobules III-VI, and crus I-II have been shown to be activated in response to painful stimuli (Moulton et al., 2010; Adamaszek et al., 2017). Interestingly, BoNT-A also significantly decreased activity in bilateral cerebellar hemispheric lobule VI, bilateral crus I, and left crus II in our patient population. Therefore, it is possible that the improvement in pain and photophobia observed in our BoNT-A responders, who had significantly higher depression scores compared to BoNT-A non-responders, may in part be attributed to BoNT-A’s effect on the cerebellum.

### 4.6. Short vs. long-acting therapies for ocular pain

Previously, we examined the impact of short term therapies (i.e., topical anesthetic) on ocular pain in individuals with photophobia (Crane et al., 2017; Choudhury et al., 2023). In the current study, we focused on a long-acting therapy, namely BoNT-A, in a similar cohort that included six individuals from the previous study (Choudhury et al., 2023). There are multiple key differences between short and long-acting treatments. In addition to chronicity of therapeutic effect, the location of administration differed between the two strategies. Topical anesthetic (proparacaine) was placed directly in both eyes, while BoNT-A was injected in the procerus, corrugator, and frontalis muscles. Additionally, the mechanisms of action are distinct. Proparacaine binds and inhibits voltage-gated sodium channels, which then stabilizes the neuronal membrane and results in a loss of sensation by inhibiting sodium ion influx (National Center for Biotechnology Information, 2023). BoNT-A acts at the neuromuscular junction to improve pain symptoms by decreasing CGRP release and reducing muscle contraction (Meng et al., 2009; Diel et al., 2019). These differences in treatment modalities may impact which neural networks are involved in pain processing and therapeutic response in individuals with chronic ocular pain and photophobia.

In both the current and previous studies, short and long-acting therapies decreased light-evoked discomfort levels at the group level (Choudhury et al., 2023). Additionally, both cohorts displayed significantly decreased BOLD activation in bilateral S1 after intervention. These highlight that regions involved with the sensory-discriminatory dimension of pain (S1) can be modulated with different treatments in individuals with chronic ocular pain and photophobia.

Differences between the short-acting proparacaine intervention and the longer-acting BoNT-A approach include a significant decrease in BOLD response in the anterior mid-cingulate cortex, a region associated with processing negative emotions linked with pain (Ploghaus et al., 2001), after topical anesthetic but not after BoNT-A injections. In contrast, regions related to the affective (bilateral S2, bilateral cerebellar hemispheric lobule VI, bilateral cerebellar crus I, and left crus II), sensory-discriminatory (bilateral S2), and motor (cerebellar vermis, and bilateral cerebellar lobule VI) dimensions of pain decreased after BoNT-A injections but not after topical anesthesia application. As pain that is unaffected by topical anesthesia is clinically defined as neuropathic (Rosenthal et al., 2009), the results of BoNT-A treatment not observed with topical anesthesia may represent therapeutic effects more closely related to changes in neuropathic pain processing. These differences highlight the heterogeneity that exists in both neural mechanisms of photophobia and chronic ocular pain as well as the mixed response to therapies in patient populations.

### 4.7. Limitations

Multiple limitations need to be considered regarding our results. First, our findings are from a small cohort with differences in demographics and co-morbidities. Importantly, patients rarely present with a single condition, making an isolated analysis of chronic ocular pain and photophobia challenging. We acknowledge that the co-morbid neurological conditions found in our cohort may have impacted our findings. For example, three participants (all responders) had a history of migraine, with one subject reporting an active migraine episode during the fMRI scan. Prior studies have found activation of the trigeminal system in patients with migraine during an active episode, including within the trigeminal nucleus (Burstein and Jakubowski, 2005; Noseda and Burstein, 2013). In addition to trigeminal neural networks, other studies have found active regions within anterior cingulate and secondary somatosensory cortices in individuals with migraine, both during an acute pain episode and in the inter-ictal period (Schwedt et al., 2015), which may have influenced our findings. Also, co-morbid ocular surface abnormalities, mood disorders, and medication regimens may have also influenced results. For example, the use of systemic pain medications and mood modulators may have influenced unpleasantness ratings. This points to the need for future studies with larger sample sizes that can better stratify individuals and explore these concepts, including individuals with primarily nociceptive contributors to ocular pain (e.g., pain in the setting of pterygium or Salzmann nodule) for comparison. Second, our protocol used an event-related model of light stimuli. Therefore, the observed significant BOLD activated regions may be related to acute pain processing in addition to, or rather than, regions associated with chronic ocular pain and photophobia. However, by choosing light as our stimulus, we targeted systems related to photophobia rather than symptomatology found in disparate neurological conditions. Third, future studies can expand on our initial analysis, which focused on significant BOLD activity before and after BoNT-A treatment. For example, an examination of relationships between activated brain regions may add to our understanding of neural mechanisms of ocular pain (Noseda and Burstein, 2013).

### 4.8. Impact

Despite these limitations, our results are important as photophobia can severely impact quality of life. Currently, methods to distinguish significant contributors to photophobia do not exist to determine the best treatment approaches. This study adds to the limited literature on the effects BoNT-A injections in individuals with photophobia and chronic ocular pain. Overall, the response to BoNT-A injections in our cohort emphasizes that there is heterogeneity in photophobia neural pathways and adds to our understanding of this complex topic.

## 5. Conclusion

Our study shows that BoNT-A injections can improve photophobia symptoms in select individuals. The variability of BoNT-A’s effects on both subjective symptoms and BOLD activity on fMRI suggests that heterogeneity exists within photophobia neural mechanisms. While our study provides useful insight into the underlying pathophysiology of chronic ocular pain and photophobia, further studies are needed to ultimately develop precision-based therapies in the future. However, our findings can be immediately implemented as BoNT-A is available for use and can be considered in appropriate patients. Based on prior reports, its effects are expected to last approximately 3 months (Silberstein et al., 2000). Therefore, if a patient reports subjective improvement after BoNT-A treatment, repeat injections every 3 months could be considered.

## Data availability statement

The datasets presented in this article are not readily available because Strict security and privacy rules regarding Veterans Affairs participants. Requests to access the datasets should be directed to eric.moulton@childrens.harvard.edu.

## Ethics statement

The studies involving human participants were reviewed and approved by The Miami Veterans Affairs (VA) and the University of Miami Institution Review Boards (IRB approval #3011.08 and 20190340, respectively). The patients/participants provided their written informed consent to participate in this study.

## Author contributions

NR was most involved in the project’s data acquisition, analysis, interpretation, and revision of the manuscript with significant assistance and input from JH, AC, NP, EL, EF, PP, AG, and EM. EF, AG, and EM significantly contributed to the study’s conception and its design. All authors provided significant contributions to this manuscript and approved the final version.

## Funding

This study was supported by the Department of Veterans Affairs, Veterans Health Administration, Office of Research and Development, Clinical Sciences R&D (CSRD) I01 CX002015 (AG, EF, and EM). This study was also supported by Department of Veterans Affairs, Veterans Health Administration, Office of Research and Development, Biomedical Laboratory R&D (BLRD) Service I01 BX004893 (AG), Rehabilitation R&D (RRD) I21 RX003883 (EF and AG), Department of Defense Gulf War Illness Research Program (GWIRP) W81XWH-20-1-0579 (AG) and Vision Research Program (VRP) W81XWH-20-1-0820 (AG and EF), National Eye Institute U01 EY034686 (EM, EF, and AG), R61EY032468 (AG), and NIH Center Core Grant P30EY014801 (institutional) and Research to Prevent Blindness Unrestricted Grant GR004596-1 (institutional).

## Conflict of interest

The authors declare that the research was conducted in the absence of any commercial or financial relationships that could be construed as a potential conflict of interest.

## Publisher’s note

All claims expressed in this article are solely those of the authors and do not necessarily represent those of their affiliated organizations, or those of the publisher, the editors and the reviewers. Any product that may be evaluated in this article, or claim that may be made by its manufacturer, is not guaranteed or endorsed by the publisher.
