# Supplementary material for: Botulinum toxin A decreases neural activity in pain-related brain regions in individuals with chronic ocular pain and photophobia
Source: Front Neurosci. 2023 Jun 19;17:1202341. doi: 10.3389/fnins.2023.1202341 (PMC10315909; doi:10.3389/fnins.2023.1202341)
Supplement: Supplementary file 1 [file Data_Sheet_1.docx]

Supplementary Material

**Botulinum Toxin A Decreases Neural Activity in Pain-Related Brain Regions in Individuals with Chronic Ocular Pain and Photophobia**

**Nicholas Reyes MD, MS; Jaxon J. Huang; Anjalee Choudhury MD; Nicholas Pondelis; Elyana V. Locatelli; Elizabeth R. Felix PhD; Pradip M. Pattany PhD; Anat Galor MD, MSPH; Eric A. Moulton OD, PhD^*^**

*** Correspondence:** Eric Moulton: [eric.moulton@childrens.harvard.edu](mailto:eric.moulton@childrens.harvard.edu)

# Supplementary Table 1. Whole Brain Group-level of Light-related Activation in Subjects.

# Brain regions with significant light-evoked activation for pre- and post-BoNT-A injection conditions, and significant decreases in light-evoked activation before and 4-6 weeks after BoNT-A injection (pre-BoNT-A > post-BoNT-A) N=12 subjects. Individual voxel threshold z>2.3, and cluster-threshold of p<0.05. L=left; R=right; B=both; C=cortex; G=gyrus.

| Brain region | Side | z-stat | Peak MNI (mm) | | | Volume (cm^3^) | Incorporated brain regions (Harvard-Oxford Cortical Atlas) |
| --- | --- | --- | --- | --- | --- | --- | --- |
|  |  |  | X | Y | Z |  |  |
| Pre-BoNT-A | | | | | | | |
| Intracalcarine C | B | 17.5 | 2 | -64 | 8 | 305.2 | Supracalcarine C, Temporal Occipital Fusiform, Cuneal C, Lingual G, Precuneous C, Lateral Occipital C, Occipital fusiform G, Cingulate G |
| Superior Parietal Lobule | L | 4.6 | -20 | -58 | 74 | 7.3 | Lateral Occipital C, Precuneous C, Postcentral G |
| Superior Parietal Lobule | L | 4.6 | -38 | -50 | 62 | 2.1 | Supramarginal G |
| Temporal Pole | L | 6.0 | -32 | 24 | -28 | 1.8 | Frontal Orbital C |
| Post-BoNT-A | | | | | | | |
| Intracalcarine C | B | 17.7 | 18 | -64 | 8 | 130.9 | Supracalcarine C, Lingual G, Cingulate G, Occipital Pole, Precuneous C, Cuneal C |
| Juxtapositional Lobule C | B | 6.5 | 0 | 6 | 64 | 20.7 | Superior Frontal G, Paracingulate G, Cingulate G |
| Inferior Frontal G | R | 6.0 | 50 | 24 | -4 | 9.6 | Frontal Operculum C, Temporal Pole, Frontal Orbital C, Insular C |
| Inferior Frontal G | L | 6.0 | -54 | 14 | 4 | 9.3 | Precentral G, Frontal Operculum C, Temporal Pole, Insular C, Frontal Orbital C, Central Opercular C |
| Precentral G | R | 5.8 | 46 | 10 | 34 | 4.2 | Middle Frontal G, Inferior Frontal G |
| Frontal Pole | R | 4.5 | 28 | 56 | 28 | 4.1 | - |
| Precentral G | L | 5.2 | -42 | 2 | 44 | 3.2 | Middle Frontal G |
| Lateral Occipital C | L | 3.9 | -32 | -62 | 54 | 2.5 | Superior Parietal L, Angular G, Supramarginal G |
| Supramarginal G | L | 4.4 | -58 | -50 | 32 | 2.5 | Angular G, Parietal Operculum C, Planum Temporale |
| Frontal Pole | L | 5.1 | -34 | 52 | 24 | 1.9 | - |
| Contrast (Pre-BoNT-A > Post-BoNT-A) | | | | | | | |
| Temporal Occipital Fusiform C | L | 5.0 | -36 | -54 | -20 | 30.3 | Inferior Temporal G, Occipital Fusiform G |
| Precentral G | L | 5.8 | -34 | -24 | 70 | 12.4 | Postcentral G, Superior Parietal Lobule |
| Occipital Fusiform G | R | 5.3 | 26 | -80 | -16 | 7.8 | Lateral Occipital C, Occipital Pole |
| Central Opercular C | L | 4.4 | -60 | -20 | 12 | 7.2 | Planum Temporale, Parietal Operculum C, Heschl’s G |
| Middle Frontal G | R | 6.0 | 38 | 2 | 64 | 6.3 | Precentral G, Superior Frontal G, Postcentral G |
| Temporal Pole | R | 5.2 | 36 | 22 | -24 | 5.6 | Frontal Orbital C |
| Middle Temporal G | R | 4.1 | 68 | -34 | 0 | 2.6 | Superior Temporal G, Planum Temporale |
| Lingual G | L | 4.0 | -12 | -62 | -6 | 1.9 | Occipital Fusiform G |
