# Supplementary material for: Botulinum toxin A decreases neural activity in pain-related brain regions in individuals with chronic ocular pain and photophobia
Source: Front Neurosci. 2023 Jun 19;17:1202341. doi: 10.3389/fnins.2023.1202341 (PMC10315909; doi:10.3389/fnins.2023.1202341)
Supplement: Supplementary file 2 [file Data_Sheet_2.docx]

Supplementary Material

**Botulinum Toxin A Decreases Neural Activity in Pain-Related Brain Regions in Individuals with Chronic Ocular Pain and Photophobia**

**Nicholas Reyes MD, MS; Jaxon J. Huang; Anjalee Choudhury MD; Nicholas Pondelis; Elyana V. Locatelli; Elizabeth R. Felix PhD; Pradip M. Pattany PhD; Anat Galor MD, MSPH; Eric A. Moulton OD, PhD^*^**

*** Correspondence:** Eric Moulton: eric.moulton@childrens.harvard.edu

# Supplementary Text 1: fMRI Acquisition and Preprocessing

Imaging was conducted using a 3T Siemens MAGNETOM Vida scanner (Erlangen, Germany) with a Siemens BioMatrix Head/Neck 20 channel coil. For anatomical scans, a sagittal three-dimensional T1-weighted scan (MPRAGE) was performed (TE/TR = 2.38/2100 ms; 192 1.00 mm-thick sagittal slices; in-plane resolution = 1.00 x1.00 mm [256 x 256]). For the functional scan, a gradient echo (GE) echo planar imaging (EPI) sequence was performed (TE/TR = 30/2000 ms; 100 1.50 mm-thick oblique slices aligned to the long axis of the caudal brainstem; in-plane resolution = 1.94x1.94 mm [136 x 136]), with 290 volumes (9 minutes and 40 seconds) captured. The oblique orientation of acquisition has proven useful for functional imaging of brainstem structures.(1) Functional imaging datasets were processed and analyzed using FEAT (FMRI Expert Analysis Tool) Version 6.0, part of FSL 6.0.0 (FMRIB's Software Library, www.fmrib.ox.ac.uk/fsl).(2) Pre-processing included elimination of the first three acquired volumes to allow for signal equilibration; motion correction using MCFLIRT (Motion Correction using FMRIB's Linear Image Registration Tool)(3); removal of non-brain structures using BET [Brain Extraction Tool](4); spatial smoothing using a Gaussian kernel of 5-mm full-width half-maximum; grand-mean intensity normalization of the entire 4D dataset by a single multiplicative factor; and high-pass temporal filtering (Gaussian-weighted least-squares straight line fitting with sigma = 50.0 s).

Time-series analysis using the general linear model was implemented with FILM (FMRIB's Improved Linear Model) with local autocorrelation correction.(7) The hemodynamic response was modeled using a gamma convolution (SD = 3 s; mean lag = 6 s) of the stimulation block.

*Whole brain processing*: Functional volumes were normalized to the Montreal Neurological Institute (MNI152) brain template for whole brain analyses of subjects at group-level and within-group. Whole brain group activation maps were created by FEAT. Fixed effects contrasts were generated using FEAT’s FLAME (FMRIB’s Local Analysis of Mixed Effects) to investigate for changes across MRI sessions, before and 4-6 weeks after BoNT-A. The time between the first and second MRI sessions ranged between 4-22 weeks. The locations of results were identified using the Harvard-Oxford Subcortical and Cortical atlases implemented within FSL.

**References**

1. Moulton EA, Becerra L, Borsook D. An fMRI case report of photophobia: activation of the trigeminal nociceptive pathway. Pain. 2009;145(3):358-63.

2. Smith SM, Jenkinson M, Woolrich MW, Beckmann CF, Behrens TE, Johansen-Berg H, et al. Advances in functional and structural MR image analysis and implementation as FSL. Neuroimage. 2004;23 Suppl 1:S208-19.

3. Jenkinson M, Bannister P, Brady M, Smith S. Improved optimization for the robust and accurate linear registration and motion correction of brain images. Neuroimage. 2002;17(2):825-41.

4. Smith SM. Fast robust automated brain extraction. Human Brain Mapping. 2002;17(3):143-55.

5. McKeown MJ, Makeig S, Brown GG, Jung TP, Kindermann SS, Bell AJ, et al. Analysis of fMRI data by blind separation into independent spatial components. Hum Brain Mapp. 1998;6(3):160-88.

6. Griffanti L, Douaud G, Bijsterbosch J, Evangelisti S, Alfaro-Almagro F, Glasser MF, et al. Hand classification of fMRI ICA noise components. Neuroimage. 2017;154:188-205.

7. Woolrich MW, Ripley BD, Brady M, Smith SM. Temporal Autocorrelation in Univariate Linear Modeling of FMRI Data. NeuroImage. 2001;14(6):1370-86.
